# Assessing sickness behavior in the French: Validation of the French translation of the sickness questionnaire (SicknessQ) in a non-clinical French population

**DOI:** 10.1016/j.bbih.2023.100708

**Published:** 2023-11-16

**Authors:** Anna Andreasson, Arnaud Tognetti, Mike Jones, Mats Lekander, Julie Lasselin

**Affiliations:** a Stress Research Institute, Department of Psychology, Stockholm University, Stockholm, Sweden; b Division of Psychology, Department of Clinical Neuroscience, Karolinska Institutet, Stockholm, Sweden; c Department of Psychology, Macquarie University, North Ryde, NSW, Australia; d CEE-M, CNRS, INRAE, Institut Agro, University of Montpellier, Montpellier, France; e Osher Center for Integrative Health, Department of Clinical Neuroscience, Karolinska Institutet, Stockholm, Sweden

**Keywords:** Sickness behavior, Questionnaire, French, Fatigue, Pain, Mood

## Abstract

The Sickness Questionnaire (SicknessQ) is a questionnaire developed to assess symptoms of sickness behavior, including somatic, behavioral, and affective dimensions. To promote cross-cultural assessments of sickness behavior, we aim to expand the use of this questionnaire to other populations and languages. The aim of the present study was to evaluate the French translation of SicknessQ in a French-speaking general population during the COVID-19 pandemic. One hundred and thirty-nine individuals completed the SicknessQ online, along with the construct criteria measures of self-rated health, state anxiety (STAI-S), and depressive symptoms (PHQ-9). The principal component analyses revealed two components: the first component included seven items concerning mood, motivation and experiences of fatigue and pain; the second component included three items concerning somatic sickness symptoms. Higher scores on the total scale and the two component subscales were associated with poorer self-rated health and higher STAI-S and PHQ-9 scores. Since the associations with construct criteria variables were relatively similar between the single- and the two-dimensional solutions, both the total scale and the subscales of the two components of the French SicknessQ can be used in future studies to measure sickness behavior in French-speaking populations.

## 1 Introduction

*Sickness behavior* refers to manifest behavioral changes and subjective experiences occurring in sick individuals (Lasselin, 2021), and includes fatigue, altered motivation for social interactions and physical activities, reduced appetite, bodily pain, feeling anxious and depressed, and cognitive impairments such as concentration difficulties. Sickness behavior is triggered by inflammatory cytokines released by activated immune cells during an infection through their effect on the central nervous system (Dantzer et al., 2008). Sickness behavior is observed across a variety of species, including insects, fishes, birds, rodents, and humans (Lasselin et al., 2020; Lopes et al., 2021), supporting adaptive properties of these behavioral changes (Hart, 1988).

The study of sickness behavior is crucial for understanding symptoms and sequela of infectious and inflammatory disorders. Of relevance in the surge after the COVID-19 pandemic, this includes the study on how sick individuals behave with consequences for the management of future epidemics (Bouayed and Bohn, 2021; Prather et al., 2020) as well as the understanding of persistent neuropsychiatric symptoms post-infection (Taquet et al., 2021; Ziauddeen et al., 2022). Furthermore, studying how sickness is detected by others (Axelsson et al., 2018; Hansson et al., 2023; Regenbogen et al., 2017; Sarolidou et al., 2019) may provide insights regarding how sickness behavior impacts social interactions between sick and healthy individuals (Smith and Bilbo, 2021) including both avoidance and stigmatization of sick individuals and promoted caregiving (Brown et al., 2022; Dantzer, 2021). Importantly, investigating the mechanisms underlying the effect of inflammatory cytokines on affective and behavioral symptoms is highly relevant to further understand inflammation-associated depression (Dooley et al., 2018; Lasselin et al., 2021; Savitz and Harrison, 2018). All of these situations require a validated assessment tool for sickness behavior.

The Sickness Questionnaire (SicknessQ) was developed in Swedish as an assessment tool to measure subjective experiences of sickness behavior in humans (Andreasson et al., 2018), and has subsequently been translated and validated in English (Andreasson et al., 2020) and Chinese (Tang et al., 2022). The SicknessQ is the only questionnaire, to the best of our knowledge, which assesses sickness behavior across various dimensions, including somatic, behavioral, and affective aspects of the subjective sickness experience. Sickness behavior indeed includes various aspects (Lasselin, 2021), and it has been suggested that somatic aspects and emotional aspects have different kinetics in response to inflammation, suggesting distinct underlying mechanisms (Capuron and Miller, 2011; Dantzer et al., 2008).

The SicknessQ has been found to be useful in both experimental (Lasselin et al., 2018) and clinical research (Astrom et al., 2022; Hedman-Lagerlof et al., 2017; Jonsjö et al., 2020; Lindsäter et al., 2018, 2023). With this questionnaire, we described the kinetic of sickness behavior changes over time in response to acute inflammation, and assessed potential underlying psychophysiological mechanisms of inflammation-induced sickness feelings (Balter et al., 2023; Lasselin et al., 2018). In clinical populations, the SicknessQ provides a tool to describe and characterize the symptomatic profile of patients and their associations with biological markers (Hedman-Lagerlof et al., 2017; Jonsjö et al., 2020; Lodin et al., 2017). Furthermore, this tool allows to measure acute changes in sickness feelings, and is thus well suited for longitudinal studies with repeated measurements (Moriarity and Slavich, 2023).

To promote validated multicultural assessments of sickness behavior, we aim to extend its use to other populations and languages. Because of a strong presence of psychoneuroimmunology and immunopsychiatry research in France (Leboyer et al., 2016), we aimed to evaluate the French translation of the SicknessQ, examining its psychometric properties, including factor structure and criteria validity, in a French speaking general population during the COVID-19 pandemic.

## 2 Methods

### 2.1 Participants and procedure

Participants were approached in March-April 2021 through multiple channels. First, we invited 800 individuals from a database of volunteers who had registered to participate in experimental research at the Laboratory of Experimental Economics in Montpellier (LEEM, University of Montpellier, France), to complete our online questionnaire. Subsequently, we distributed the survey link through the national database, Risc (Relais d’Information sur les Sciences de la Cognition, https://expesciences.risc.cnrs.fr), which comprised more than 12,000 volunteers. Finally, we spread the link via social media. Participation was voluntary and non-remunerated. Only participants who were older than 18 and fluent in French were asked to participate. Participants were told that they would be asked to complete a set of questionnaires on topics linked to sickness and infections, and signed a consent before starting the survey. The survey took 15–20 min to complete and responses were anonymous.

Questionnaire data were collected using REDCap (Research Electronic Data Capture) hosted at Karolinska Institutet, Sweden (Harris et al., 2009, 2019), which is a secure platform designed to collect data for research studies. Two quality checks were included (“it is important that you read every question, please do NOT answer 3”, and “it is important that you read every question, please choose the answer 3”) and the data were used only if participants passed the two quality checks.

A total of 219 participants answered the survey, but 55 of them did not complete the survey and their data were thus not included. Furthermore, 19 participants did not meet the required quality standards. Of the remaining 145 participants, six reported having only an intermediate or advanced proficiency in French and were therefore excluded from the analyses. Ultimately, data from 139 participants were analyzed. The average age of these participants was 38 years (*SD* = 17 years), ranging from 18 to 85. Among them, 99 (71%) were women, 37 (27%) were men, two self-identified as non-binary, and one preferred not to disclose their gender. As it was mandatory to complete all items of the survey, no data was missing for the included participants.

Study data are available at https://osf.io/x69j2/?view_only=82b9a879e8064edfad8f4a651d9acf9a.

### 2.2 SicknessQ

The Sickness Questionnaire includes 10 statements of sickness feelings (“I want to keep still”, “My body feels sore”, “I wish to be alone”, “I don't wish to do anything at all”, “I feel depressed”, “I feel drained”, “I feel nauseous”, “I feel shaky”, “I feel tired”, “I have a headache”; see Table 1 for the corresponding French translation) rated on a 4-point scale from disagree (0) to agree (3) (Andreasson et al., 2018). The 10 items are summed to provide a score from 0 (no sickness behavior) to 30 (very intense sickness behavior).

**Table 1:** Principal Component Analyses of the SicknessQ items.

Item	Three components			Two components	
	1	2	3	1	2
1. I want to keep still *Je ne veux pas bouger*	**.774**	−.215	.171	**.604**	−.426
2. My body feels sore *Mon corps est douloureux*	−.142	−.255	**−.852**	**.325**	.124
3. I want to be alone *Je souhaite être seul·e*	**.585**	.110	.184	**.441**	−.094
4. I don't wish to do anything at all *Je n'ai rien envie de faire du tout*	**.712**	−.073	−.356	**.844**	−.080
5. I feel depressed *Je me sens déprimé·e*	**.644**	.119	−.356	**.791**	.110
6. I feel drained *Je suis lessivé·e*	.404	.329	**−.462**	**.640**	.393
7. I feel nauseous *J'ai la nausée*	−.068	**.876**	.128	−.090	**.767**
8. I feel shaky *Je me sens faible et tremblant·e*	−.018	**.836**	−.099	.079	**.807**
9. I feel tired *Je suis fatigué·e*	.333	.207	**−.636**	**.664**	.363
10. I have a headache *J'ai mal à la tête*	−.048	.204	**−.537**	.260	**.403**

Loading values for each PCA component of the SicknessQ. Each item was deemed loading into a specific component if: 1) the loading value was higher than 0.3; and 2) the loading value was the highest in this component for this item.

The questionnaire was originally developed in Swedish, and included items that were responsive to experimentally-induced sickness behavior, and psychometric properties and criteria validity was evaluated in a primary care population. Here, the English version of the SicknessQ, validated previously (Andreasson et al., 2020), was translated into French by two independent bilingual native French speakers and the resulting translation is the consensus of the two independent translations. The English version was used as the basis for the French translation since the French speakers were not fluent in Swedish. The questionnaire was then translated back into (1) Swedish by two independent bilingual native Swedish speakers, and (2) English by one bilingual native English speaker, to ensure that the meaning had not been altered during the translation process. After the back-translation, three items for which the back-translation did not correspond to the original meaning were discussed to find the best translation: “I want to keep still” (item 1) was originally translated to “je veux rester tranquille” and back translated to “I want to stay calm”, and thus changed to “je ne veux pas bouger”; “I feel drained” (item 6) was originally translated to “je me sens extenué·e” and back translated to “I feel extremely weak”, and thus changed to “Je suis lessivé·e”; “I feel shaky” (item 8) was originally translated to “Je tremble” and back translated to “I am shivering”, and thus changed to “Je me sens faible et tremblant·e".

### 2.3 Criteria variables

**Self-rated health** was assessed using the question “How do you rate your general state of health?” rated on a 5 point scale from very good (1) to very poor (5). **State anxiety** was assessed using the state part of the State Trait Anxiety Inventory (STAI-S) (Spielberger et al., 1979), which contains 20 items and scores the intensity of state anxiety symptoms on a scale from 20 to 80. Cronbach's alpha for STAI-S was 0.95. **Depressive symptoms** were assessed using the Patient Health Questionnaire- 9 (PHQ-9) (Kroenke et al., 2001), scoring the intensity of depressive symptoms over the past two weeks on a scale from 0 to 27. Cronbach's alpha for PHQ-9 was 0.88.

### 2.4 Statistics

The French SicknessQ was validated through a three-step process, after our preliminary confirmatory factor analysis (CFA), which did not meet all of the necessary model fit criteria for a single factor model in the present sample. First, we performed a principal components analysis (PCA) of the 10 items to determine the best item structure, together with a parallel analysis to compare the eigenvalues obtained in the PCA with random eigenvalues (O'connor, 2000). Second, in order to quantify how well the proposed item structure was supported by the data, we conducted CFAs using both the 10-item single-factor solution from the original validation and the item structures established through the PCA. It is important to note that this use of CFA does not constitute a validation of our model since it is fitted to the same data as used to develop the model. The best model was selected based both on CFA criteria and on the theoretically soundness of the factor solution (i.e. consistent with the existing literature on sickness behavior). Finally, we assessed the correlation of the total score and the scores from both the single- and dual-factor solutions with criteria validity variables. All analyses, apart from CFAs, were conducted using IBM SPSS Statistics 27. As per our internal quality control process, the analyses were repeated by an independent investigator using R 4.1.3 (figures from SPSS are reported). CFA analyses were conducted using STATA 17.

#### 2.4.1 Principal component analyses

The 10 items of the SicknessQ were included in the PCA using an oblimin rotation to allow correlated components. Since the aim was to identify strong patterns of correlations among all items and to reduce the dimensionality of the SicknessQ (rather than to obtain latent variables that explains variability in the items), PCA was deemed more appropriate than Exploratory Factor Analysis (EFA).

We tested both a 2-component solution and a 3-component solution because of the risk for the PCA to produce more components than actually exist, because of the eigenvalue of the second component (1.55) being close to the eigenvalue of the third component (1.24), and to determine if the resulting components would fit better the theoretical knowledge of sickness behavior (Dantzer et al., 2008).

An eigenvalue cut-off of 1 was employed to identify the latent component(s). Furthermore, we conducted a parallel analysis (O'connor, 2000), to compare the eigenvalues obtained in the PCA with random eigenvalues obtained with the parallel analysis. The number of components is confirmed by the parallel analysis if the 95th percentile interval of the random eigenvalues are lower than the eigenvalues obtained in the PCA.

#### 2.4.2 Confirmatory factor analysis

The single, two-, and three-component models from the PCAs were evaluated in CFA to quantify which was better supported by the data. Covariance terms (i.e. covariance between residuals of observed items) which would improve model fit substantively (reduction in residual Chi-Square statistic >10) were included as long as they did not alter the a priori specified item structure. Several metrics including the residual Chi-Square test (ideally p > 0.05), the ratio of Chi-Square to degrees of freedom (ideally <5.0), the comparative fit index (CFI, ideally >0.95) and the Tucker-Lewis index (TLI, ideally >0.95) and root mean square error of approximation (RMSEA), ideally <0.05 were used (Schermelleh-Engel et al., 2014) based on the recommended criteria (Schermelleh-Engel et al., 2003). Akaike information criterion (AIC) was used to compare fit between models.

#### 2.4.3 Associations with criteria variables

Associations of the total score and the subscores based on the two-component solution (calculated using the sum of the items included in each component, with higher score indicating stronger sickness behavior) with the criteria variables were assessed using multiple linear regressions. The independent variables included self-rated health, PHQ-9 total score, and STAI-S total score, while age and gender were entered as covariates. The dependent variable was the total SicknessQ score or the subscore for the first factor. Because residuals of the regression models with the Component 2 of the 2-dimensional solution were not normally distributed, non-parametric Spearman correlations were conducted to assess the association between the subscore of the second factor and the criteria variables. Data from the two non-binary participants and the one who did not want to report gender were excluded from these analyses.

#### 2.4.4 Statistical power

A sample of n = 110 provides statistical power 0.8 at the 0.01 level of statistical significance, for a model with five criteria variables simultaneously, and setting incremental explained variance of 10% (ΔR^2^ = 0.1) for any criterion variable as the minimum important effect size. The actual sample size recruited exceeds this requirement and provides slightly higher power than required.

## 3 Results

The average SicknessQ total score was 6.22 (*SD* = 4.58) with a range of 0 (minimum) and 20 (maximum). The average PHQ9 score was 7.73 (*SD* = 5.93, range 0–25) and average STAI-S score was 41.4 (*SD* = 12.8, range 20–79). Among the 139 participants, 18 (12.9%) had a PHQ-9 score between 10 and 14 (cut-off for moderate depressive symptoms), 16 (11.5%) had a PHQ-9 score between 15 and 19 (cut-off for moderately-severe depressive symptoms), and 8 (5.8%) had a PHQ-9 score between 20 and 27 (cut-off for severe depressive symptoms). Sixty-seven (48.2%) participants had a STAI-S score of 41 or above (cut-off for clinical levels of anxiety symptoms). The average self-rated health was 2.19 (*SD* = 0.79). Among the 139 participants, 22 (15.8%) rated their health as very good, 78 (56.1 %) rated their health as good, 31 (22.3%) rated their health as neither good nor bad, 7 (5%) rated their health as bad and 1 (1%) rated their health as very bad.

### 3.1 Principal component analyses

**Fig. 1:**
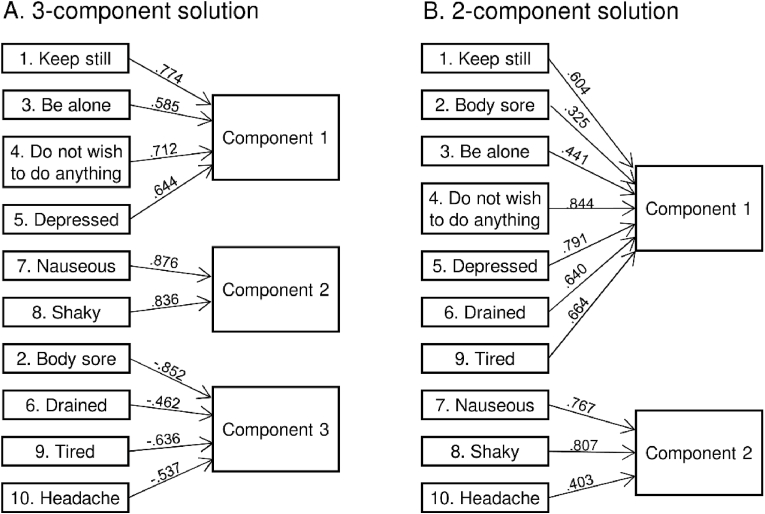
**Illustration of the 3-component and 2-component solutions of the PCA.** Values indicate the loading values for each item in the respective component.

The PCA based on the 10 SicknessQ items suggested a 3-dimensional solution (Table 1, Fig. 1). Component 1 included emotional and motivational items: *I want to keep still, I want to be alone, I don't wish to do anything at all,* and *I feel depressed.* Component 2 included the somatic-oriented items *I feel nauseous,* and *I feel shaky.* Component 3 included items related to pain and fatigue, which were all negatively loaded (higher values in this factor thus indicate lower intensity of symptoms): *My body feels sore, I feel drained, I feel tired,* and *I have a headache*. Each of the three factors explained respectively 34%, 16%, and 12% of the variance.

We also tested a 2-dimensional solution (Table 1, Fig. 1). Component 1 explained 34% of the variance and included seven items concerning emotion, motivation, fatigue, and widespread pain: *I want to keep still, My body feels sore, I want to be alone, I don't wish to do anything at all, I feel depressed, I feel drained,* and *I feel tired.* Component 2 explained 16% of the variance, and included items concerning somatic sickness symptoms including one on localized pain: *I feel shaky, I feel nauseous,* and *I have a headache*.

The parallel analysis supported the 2-dimensional solution. The 95% percentiles of the eigenvalues for the three first random components provided by the parallel analysis were: 1.63, 1.39, 1.27. The eigenvalues for the first two components in the PCA were 3.41 and 1.55, which are higher than the first two 95th percentiles of the random components. However, the eigenvalue of the third component was 1.24, which is close but lower than the third 95th percentile of the random components, suggesting that the third component obtained in the PCA was not different than chance.

### 3.2 Confirmatory factor analyses

The 2-dimensional solution including covariance terms fulfilled all criteria for adequate fit apart from RMSEA of 0.051, while the 3-dimensional solution did not (see Table 2). The improvement in AIC between the 3- and 2-dimensional solution was 0.017%, and 0.70% between the single- and 2-dimensional solution. In addition to being simpler than the 3-dimensional solution, the components in the 2-dimensional solution provides a better theoretical fit (see Discussion). Hence, the 2-dimensional solution together with the original single factor solution were further evaluated against construct criteria variables.

**Table 2:** Fit statistics of the single, two, and three component models of SicknessQ.

	Single	2-dimensional	3-dimensional
Chi^2^/df	1.95	1.27	1.42
p > chi2	0.001	0.094	0.035
RMSEA	0.083	0.051	0.061
AIC	2874.413	2856.636	2861.593
BIC	2971.251	2959.342	2964.299
CFI	0.923	0.973	0.961
TLI	0.892	0.960	0.941

*Abbreviations:* RMSEA: Root mean square error of approximation; AIC: Akaike's information criterion; BIC: Bayesian information criterion; CFI: Comparative fit index; TLI: Tucker–Lewis index.

### 3.3 Associations with the criteria variables

Higher scores on the total scale and the two subscales were associated with poorer self-rated health and higher STAI-S and PHQ-9 scores (Table 3) supporting the criterion validity of the instrument.

**Table 3:** Association of the SicknessQ total and component scores with the criteria variables.

	SicknessQ Total score (0–30) $\bar{x}$ = 6.22, *SD* = 4.58			SicknessQ Component 1 (0–21) Affective-fatigue subscore $\bar{x}$ = 5.63, *SD* = 4.03			SicknessQ Component 2 (0–9) Somatic subscore $\bar{x}$ = 0.58, *SD* = 1.20	
	β	P-value	95% CI	β	P-value	95% CI	ρ	P-value
SRH	0.203	.006	0.349–1.997	0.185	.012	0.213–1.665	.375	<.001
PHQ-9	0.393	<.001	0.182–0.435	0.405	<.001	0.168–0.391	.330	<.001
STAI-S	0.252	.004	0.030–0.150	0.252	.004	0.026–0.132	.354	<.001
age	−0.111	.065	−0.062–0.002	−0.119	.050	−0.056–0.000	–	–
gender	0.054	.356	−0.624–1.725	0.040	.488	−0.671–1.398	–	–

*Abbreviations:* SRH: Self-rated health; PHQ-9: Patient Health Questionnaire-9, STAI-S: State-Trait Anxiety Inventory – State part.

## 4 Discussion

The present study aimed to validate a French translation of the Sickness Questionnaire in a sample from the general population during the COVID pandemic (March-April 2021). While the original single-dimension solution demonstrated acceptable, yet imperfect, fit, we sought to explore other item structures. A two-dimensional solution was suggested since it fulfilled all criteria for model fit (apart from RMSEA) while also having good theoretical support. The two-dimensional solution indicated two subscales: one concerning emotion, motivation, and experiences of fatigue and soreness, and the other encompassing somatic aspects of sickness behavior, i.e. nausea, shivering, and headache. The improvement in AIC between the single and the two-dimensional solution was less than 1 %, meaning that both the total scale and the two subscales of the French SicknessQ is justified for use according to context. However, the two-factor solution has been developed in this sample and is in need of independent validation before it is used routinely in future research. While it has merit from a theoretical sense and some empirical support from these data, the single factor model remains the standard view of the SicknessQ.

The two subscales of the French SicknessQ theoretically fit partly with the sickness behavior literature, which has featured two dimensions in inflammation-induced symptoms (Dantzer et al., 2008). Some previous studies have suggested that the affective aspects of sickness behavior might be distinct from the neurovegetative symptoms (Capuron and Miller, 2011; Dantzer et al., 2008). Furthermore, different time-courses of the affective aspects and somatic aspects were found during acute immune activation in response to a bacterial stimulus (lipopolysaccharide, LPS): while headache and nausea reached their peak level within an hour post-LPS injection and then quickly decline to lower levels, other aspects of sickness behavior developed more slowly and persisted at high levels for at least 3 h post-injection (Lasselin et al., 2017). This might indicate partly separate central mechanisms by which various aspects of sickness behavior are triggered. It could also reflect the involvement of top-down mechanisms in addition to immune processes in the formation of the affective, but not somatic, aspects of sickness behavior (Lasselin et al., 2018). Of note, two components of “somatic” vs “mental” aspects of sickness behavior were also characterized in the Chinese version of the SicknessQ (Tang et al., 2022), although the mental component was purely related to affective items in that study.

An intriguing issue is the presence of one pain item in each of the two subscales. Specifically, “my body feels sore” is present in the affective-fatigue subscale, whereas “I have a headache” is more connected to the somatic subscale. These two items, however, reflect different types of pain. Headache during immune activation is localized and is indicated to result from vascular changes in the brain and/or activation of the brainstem nuclei by inflammatory cytokines (De Marinis and Welch, 1992). Bodily pain and soreness during immune activation, on the other hand, likely result from an increased sensitivity to stimuli that are usually not painful (hyperalgesia) (Benson et al., 2015; Karshikoff et al., 2016). Hyperalgesia results from changes in pain-responsive neurons of the spinal cord dorsal horn (Bennett, 2012) as well as changes in the interpretation of interoceptive (bodily) signals and is strongly influenced by top-down mechanisms (Buchel et al., 2014). If top-down mechanisms are involved in the summation of pain from various body sites, this process might in speculation be sensitive to emotional state and therefore more related to affective than sensory pain components. Along this line of reasoning, the item “my body feels sore” may be expected to load in the same component as emotional alterations and fatigue.

One limitation of the current study is the context during which it was conducted (COVID-19 pandemic). This might have led to the average total score of SicknessQ of this French general population (6.2) being slightly higher than the average total score of SicknessQ in a Swedish general population (5.4) (Jonsjö et al., 2020), despite being a younger population (38 years old in the present study compared to 53 years old in the Swedish study). Hence, the present population was possibly influenced by worries regarding their health development during the ongoing pandemic. This notion is also supported by the fact that self-rated health in the present population was similar to primary care populations (Lodin et al., 2017). On the other hand, sickness behavior is typically analyzed in clinical populations and in conjunction with activation of immune processes, and conducting the study in a non-clinical population during a state of constant infection threat can also be appraised as a strength. Future studies should evaluate the validity of the French SicknessQ to measure sickness behavior in French-speaking clinical populations and using experimental investigations of immune activation.

Another limitation of this study pertains to the characteristics of the sample. Despite our efforts to advertise the study to various local and national participant databases, the number of respondents was relatively low and not representative of the French general population. Indeed, our sample was predominantly composed of a relatively young population of respondents, with a bias toward women, which hinders the generalizability of the findings, although it is representative of the population of people with functional somatic syndromes (FSS) which tend to have a female predominance (Narayanan et al., 2021). Another piece of the evidence that is not provided by the current study is validation in a sample of individuals suffering from FSS, who are of particular interest in terms of the application of this instrument (Andreasson et al., 2020). Furthermore, we could only assess the association between the SicknessQ (sub-)scores with questionnaires assessing components of sickness behavior as criteria variables, since there is no other gold standard of sickness behavior measurement.

Overall, we believe that the SicknessQ is a valuable tool with relevance for various fields, from psychoneuroimmunology to human evolutionary biology and public health. The current study provides a French translation of the SicknessQ that supports valid assessments of sickness behavior in French-speaking populations. Both the total score of SicknessQ, and the affective-fatigue and somatic subscales, can provide meaningful information on sickness, pertinent to measurements of health and body perception in research as well as health care contexts. In a broader perspective, SicknessQ has practical applications in studying individual variation in sickness behavior, which can have important implications for public health. For example, certain individuals may be more susceptible to severe symptoms during infection, limiting their mobility, while others may be able to maintain normal functioning despite being infected, leading to an increased spread of pathogens. Therefore, the SicknessQ may be useful to examine inter-individual variations in how individuals respond to infections and how it can impact the spread of diseases within society.

## Funding

Julie Lasselin and Arnaud Tognetti were supported by the 10.13039/501100004359Swedish Research Council (10.13039/501100004359Vetenskapsrådet, 2020-01606 to JL, and 2021–03184 to AT).

## Declaration of competing interest

The authors declare that they have no known competing financial interests or personal relationships that could have appeared to influence the work reported in this paper.

## Data Availability

Study data are available at https://osf.io/x69j2/?view_only=82b9a879e8064edfad8f4a651d9acf9a
